# Atomic-Scale Mechanism of Titanium-Induced Frictional Removal of Silicon Carbide: A Molecular Dynamics Study

**DOI:** 10.3390/ma19173721

**Published:** 2026-08-31

**Authors:** Xu Ling, Wanbo Tian, Yan Li, Liqi Li, Junliang Dai, Kunpeng Li, Zhuo Li

**Affiliations:** 1 College of Intelligent Control Engineering, Hunan Chemical Technical University, Zhuzhou 412000, China; 2 College of Mechanical and Vehicle Engineering, Hunan University, Changsha 410082, China; 3 College of Civil Engineering, Hunan University, Changsha 410082, China; liyan@hnu.edu.cn; 4 Institute of Manufacturing Engineering, Huaqiao University, Xiamen 361021, China; 25014088015@stu.hqu.edu.cn

**Keywords:** silicon carbide (SiC), titanium, molecular dynamics simulation, friction-induced material removal, amorphization, graphitization

## Abstract

The atomic-scale mechanism of the titanium-induced frictional removal of 3C-SiC remains insufficiently understood, particularly regarding the role of interfacial chemical interactions in material removal. In this study, molecular dynamics (MD) simulations were performed to systematically investigate the interfacial reactions, atomic migration, and structural evolution during titanium friction on SiC surfaces. The results demonstrate that Ti atoms diffuse into the SiC lattice, while Si and C atoms migrate toward the Ti layer, leading to the formation and evolution of Ti–Si and Ti–C bonds at the friction interface. These tribochemical interactions disrupt the stable Si–C bonding network, promote atomic rearrangement, and accelerate lattice amorphization, thereby facilitating friction-induced material removal. Compared with the Si-terminated surface, the C-terminated surface exhibits stronger Ti–C chemical affinity, resulting in enhanced interfacial diffusion, more severe amorphization, and pronounced graphitization. Moreover, increasing the indentation depth mainly enhances mechanical deformation, whereas higher sliding velocity and temperature promote atomic activation and interfacial chemical reactions. This work reveals the synergistic mechanism of tribochemical bonding and structural transformation during titanium-assisted SiC removal, providing fundamental insights and theoretical guidance for the development of high-efficiency and low-damage ultra-precision machining technologies for SiC.

## 1. Introduction

Silicon carbide (SiC), as a representative wide-bandgap semiconductor, has become an indispensable engineering material owing to its outstanding mechanical, thermal, and electrical properties [1,2]. Its exceptional hardness (Mohs hardness of approximately 9.5), high thermal stability (melting point of about 2700 °C), excellent chemical inertness, and wide bandgap (3.2 eV) enable superior performance under high-temperature, high-frequency, and high-power operating conditions, significantly outperforming conventional silicon-based materials [3,4,5]. These unique characteristics have promoted the widespread application of SiC in advanced technologies. In the semiconductor industry, SiC power devices can improve inverter efficiency by 5–10%, making them key components in electric vehicles, smart grids, and renewable energy conversion systems [6]. In the aerospace sector, SiC is widely used in high-temperature structural components such as rocket engine nozzles [7]. Moreover, SiC-based devices have found extensive applications in photovoltaic power generation, rail transportation, and nuclear energy systems [8]. With the rapid development of semiconductor technology and intelligent manufacturing, the demand for high-quality SiC components is expected to continue growing. In addition to monolithic SiC, carbon nanostructure-reinforced 3C-SiC composites have attracted increasing attention due to their enhanced mechanical properties and structural stability. MD studies have demonstrated that carbon nanotubes, including single-walled and multi-walled nanotubes, can improve the mechanical response of 3C-SiC composites through strong interfacial bonding and load transfer mechanisms [9]. However, the enhanced hardness and structural integrity of these advanced SiC-based materials also increase the machining difficulty, highlighting the need to understand their atomic-scale material removal mechanisms.

The widespread application of SiC in precision electronic and optical devices requires excellent surface integrity and dimensional accuracy. However, its high hardness and wear resistance make ultra-precision machining extremely challenging [10,11]. Conventional grinding and polishing processes often suffer from either severe subsurface damage or low material removal efficiency. At present, the precision machining of SiC mainly relies on diamond grinding, chemical mechanical polishing (CMP), and energy-field-assisted polishing technologies [12]. Diamond grinding provides relatively high material removal rates but inevitably introduces subsurface damage due to brittle fracture. Although CMP can achieve atomically smooth surfaces, its material removal rate is generally too low for high-efficiency manufacturing [13,14]. Energy-field-assisted polishing, such as ultrasonic- or laser-assisted polishing, has been developed to improve both machining efficiency and surface quality [15,16]. Nevertheless, the introduction of additional energy fields significantly increases equipment complexity and manufacturing costs. Therefore, developing a cost-effective and high-efficiency ultra-precision machining technology for SiC remains an urgent research objective.

Transition metals, including Fe, Ti, Ni, and Cr, exhibit strong chemical affinity toward both silicon and carbon atoms and are capable of weakening the stable Si–C covalent bonds under appropriate thermomechanical conditions [17,18]. At elevated temperatures and contact pressures, these reactive metal atoms diffuse into the SiC surface and undergo interfacial chemical reactions, thereby facilitating material removal through tribochemical processes. In recent years, reactive metal-induced friction polishing has emerged as a promising technique for the precision machining of SiC. Wu et al. [19] employed pure iron to polish single-crystal 4H-SiC wafers and achieved a material removal rate of 375 nm/min together with an ultra-smooth surface roughness of Ra 2.2 nm. Their study demonstrated that friction-induced solid-state chemical reactions at the Fe/SiC interface played a dominant role in material removal. In a subsequent study, Wu et al. [20] investigated nickel-induced friction polishing on both the Si-terminated and C-terminated surfaces of 4H-SiC. They reported that the Si-terminated surface exhibited a higher material removal rate (8.9 μm/min), whereas the C-terminated surface achieved superior surface quality. Despite these experimental advances, the dynamic evolution of the metal/SiC friction interface cannot be directly characterized in situ. Consequently, the atomic-scale mechanisms governing reactive metal-induced frictional removal of SiC remain poorly understood.

MD simulation has become an effective computational approach for investigating material deformation and removal mechanisms at the atomic scale by numerically solving Newton’s equations of motion. Unlike experimental techniques, MD simulations enable real-time tracking of individual atomic trajectories and provide direct insight into atomic-scale structural evolution under coupled mechanical, thermal, and chemical effects. Recent all-atom MD studies have also demonstrated the capability of atomistic simulations to elucidate nanoscale wear and material-removal mechanisms [21]. In addition, normal dynamics has recently been proposed as an alternative atomistic simulation approach that can reduce the computational cost for large-scale dynamical simulations [22]. In studies of SiC machining, MD simulations have successfully revealed subsurface amorphization, crack initiation and propagation, phase transformations, and atomic diffusion during material removal. Noreyan et al. [23] performed MD simulations of nanoscratching on 3C-SiC and systematically investigated the dependence of scratch hardness and friction coefficient on scratching velocity, revealing the atomic-scale origin of the reduction in friction coefficient with increasing scratching speed. Xi et al. [24] established an MD model of diamond abrasive scratching on 4H-SiC and analyzed the effects of scratching depth and velocity on scratching force, temperature evolution, dislocation behavior, and subsurface damage.

In the present study, MD simulations were conducted to investigate the atomic-scale mechanisms governing the titanium-induced frictional removal of 3C-SiC. Particular attention was devoted to the interfacial atomic diffusion, chemical bonding evolution, and lattice structural transformation during the friction process. Furthermore, the influences of sliding velocity, temperature, and crystal orientation on interfacial diffusion behavior and structural evolution were systematically evaluated. The removal characteristics of the Si-terminated and C-terminated surfaces were also compared to clarify the role of crystallographic orientation in friction-induced material removal. The findings provide fundamental insights into the tribochemical removal mechanism of SiC and offer theoretical guidance for the development of high-efficiency, low-damage ultra-precision machining technologies.

## 2. MD Simulation

### 2.1. Simulation Model

**Figure 1:**
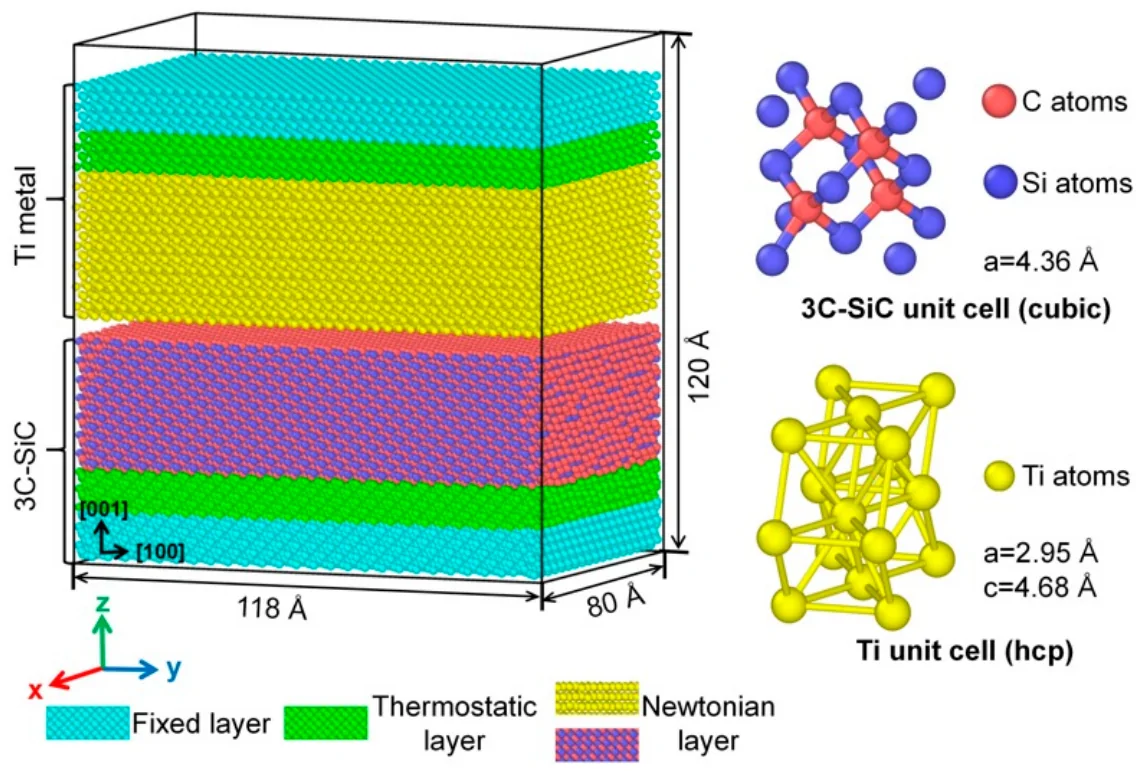
MD simulation model of titanium friction on the SiC substrate.

Classical MD simulations were performed using the Large-scale Atomic/Molecular Massively Parallel Simulator (LAMMPS) package (LAMMPS 64-bit 29Aug2024-MSMPI, USA) [25] to investigate the frictional interaction between a single-crystal titanium slider and a 3C-SiC substrate. As illustrated in Figure 1, the simulation cell had dimensions of 118 Å × 80 Å × 120 Å. Periodic boundary conditions were applied along the x- and y-directions to minimize finite-size effects, whereas a non-periodic boundary condition was imposed along the z-direction.

Titanium was modeled with a hexagonal close-packed (hcp) crystal structure, with lattice constants of a = 2.95 Å and c = 4.68 Å [26]. The substrate consisted of cubic 3C-SiC with a lattice constant of 4.36 Å [27]. In this MD model, the number of Si atoms was 23,100, the number of C atoms was 23,800, the number of Ti atoms was 28,800, and the total number of atoms was 75,700. To ensure numerical stability and control friction-induced heat accumulation during the friction process, both the titanium slider and the SiC substrate were divided into three regions: a fixed layer, a thermostat layer, and a Newtonian layer. The fixed layer constrained the atomic positions and imposed the prescribed sliding motion on the titanium slider, while the thermostat layer dissipated the friction-generated heat to maintain the system temperature within the target range. The Newtonian layer evolved freely according to Newton’s equations of motion. For the titanium slider, the thicknesses of the fixed, thermostat, and Newtonian layers were 10 Å, 10 Å, and 35 Å, respectively. For the SiC substrate, the corresponding thicknesses were 10 Å, 10 Å, and 30 Å, respectively. The Nosé–Hoover thermostat [28] was employed in the thermostat layers to regulate the system temperature while avoiding the introduction of stochastic forces that could directly perturb the atomic trajectories and interfacial atomic diffusion. The thermostat was restricted to the designated thermostat layers, whereas the Newtonian layer near the friction interface was not directly thermostatted, allowing the local atomic dynamics and structural evolution to develop naturally. The crystallographic orientation of the titanium slider was configured such that the Ti (100) plane was parallel to the 3C-SiC (100) surface, and the sliding direction was aligned with the <100> crystallographic direction of the SiC substrate.

### 2.2. Interatomic Potential Functions

The selection of an appropriate interatomic potential is critical for accurately describing atomic interactions and predicting the structural evolution of materials during MD simulations. Therefore, the reliability of the simulation results strongly depends on the choice of the potential function.

In the present study, the Tersoff/ZBL potential [29] was employed to describe the interactions among Si–Si, C–C, and Si–C atom pairs within the 3C-SiC substrate. By combining the many-body Tersoff potential with the short-range Ziegler–Biersack–Littmark (ZBL) repulsive potential, this potential accurately reproduces the crystal structure of SiC while accounting for short-range interatomic interactions under highly compressed contact conditions. Consequently, it is well-suited for modeling the large atomic deformation and collision processes occurring during friction-induced material removal. The total potential energy can be expressed as follows:

(1) $$E=\frac{1}{2}\sum_{i}\sum_{i\neq j}V_{ij}$$

(2) $$V_{ij}=(1-f_{F}(r_{ij}))V^{\mathrm{ZBL}}(r_{ij})+f_{F}(r_{ij})V^{\mathrm{Tersoff}}(r_{ij})$$

where *E* is the total potential energy, *V_ij_* represents the interatomic potential, *V*^ZBL^(*r_ij_*) denotes the ZBL potential, and *V*^Tersoff^(*r_ij_*) represents the Tersoff potential. *f_F_*(*r_ij_*) is a Fermi-type switching function that provides a smooth transition between the short-range ZBL repulsive potential and the Tersoff potential.

The Tersoff potential was employed to describe the Ti–Ti, Ti–C, and Ti–Si interactions [30]. As a many-body bond-order potential, it accounts for the effects of the local atomic environment and coordination on interatomic interactions, enabling the description of interfacial structural evolution during the Ti-induced friction process. The total potential energy is given by:

(3) $$E=\frac{1}{2}\sum_{i}\sum_{i\neq j}f_{c}(r_{ij})\left[f_{R}(r_{ij})+b_{ij}f_{A}(r_{ij})\right]$$

where *f_c_*(*r_ij_*) is the truncation function of the interaction between atoms, *f_R_*(*r_ij_*) is the potential of the attractive term, *f_A_*(*r_ij_*) is the potential of the repulsive term, *r_ij_* is the truncation radius. *b_ij_* is a lower-order function that includes the dependence on bond angles and multi-body interactions.

Although reactive force fields such as ReaxFF can explicitly describe dynamic bond breaking and formation [31], the present study primarily focuses on atomic diffusion, structural evolution, amorphization, and the interfacial interactions between Ti and SiC during frictional removal. Therefore, a combination of the Tersoff/ZBL and Tersoff potentials was adopted to balance the physical description of atomic interactions and computational efficiency. This potential scheme enables the modeling of interfacial deformation and atomic interactions during large-scale friction simulations while maintaining a manageable computational cost.

### 2.3. Simulation Procedure and Parameters

**Figure 2:**
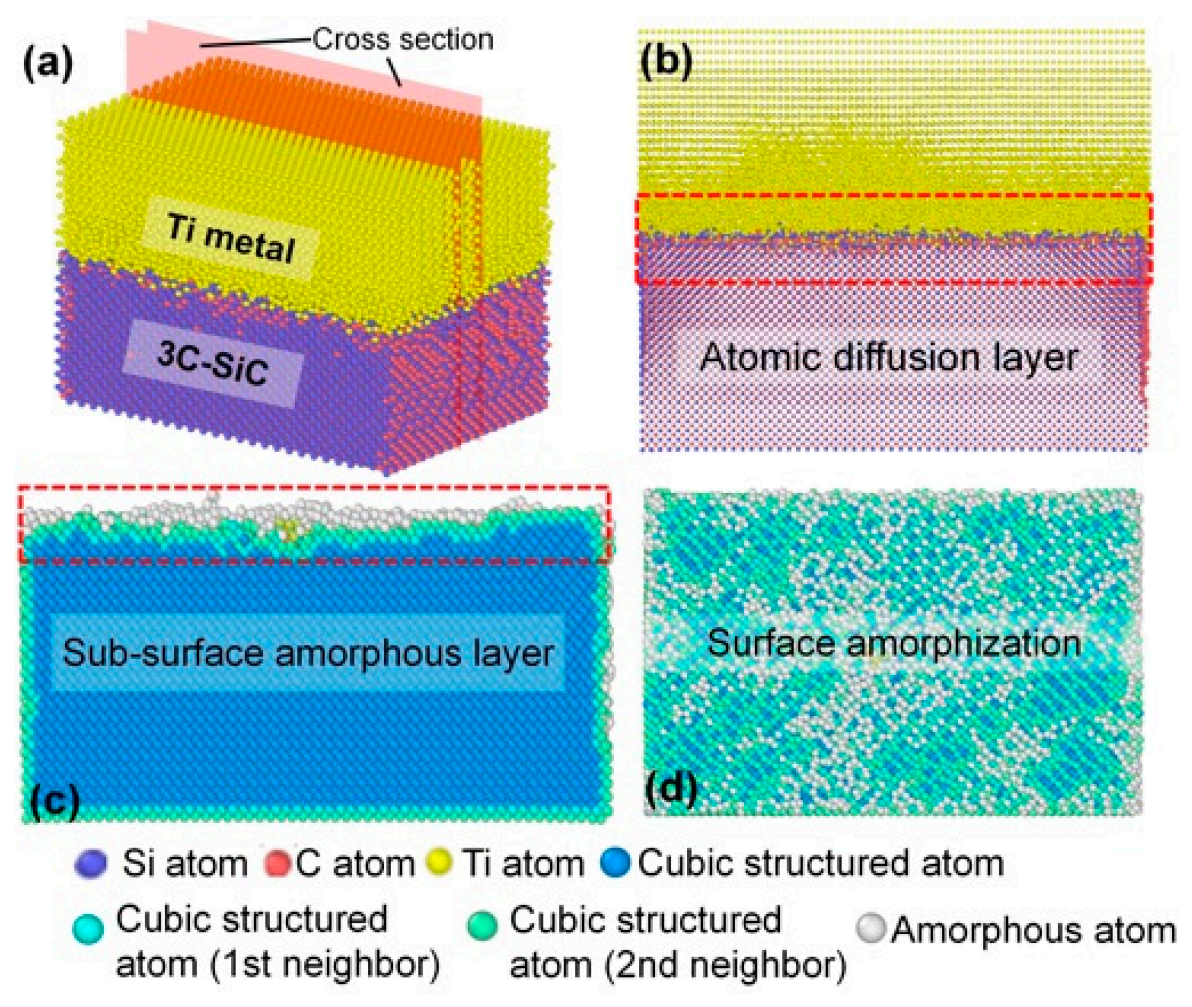
Atomic diffusion and lattice evolution during titanium friction on the Si-terminated SiC surface. (**a**) Cross-sectional configuration of the simulation model, (**b**) atomic diffusion across the Ti/SiC friction interface, and (**c**,**d**) lattice structures of the surface and subsurface regions of SiC after friction.

The simulation of titanium-induced friction on the SiC substrate consisted of three sequential stages: energy relaxation, indentation, and sliding, as illustrated in Figure 2. To eliminate artificial atomic interactions between the titanium slider and the SiC substrate during the initial relaxation process, the initial separation between the bottom surface of the titanium slider and the top surface of the SiC substrate was set to 5 Å.

Before friction simulation, the Ti/SiC system was relaxed using energy minimization to obtain a stable initial configuration. The relaxed system was then equilibrated under the canonical ensemble (NVT) at the target temperature. During friction, the sliding motion was generated by applying a constant velocity to the fixed layer of the Ti slider, while the bottom fixed layer of the SiC substrate was constrained. The thermostat layers were maintained using the Nosé–Hoover thermostat, and the Newtonian layers were evolved under the microcanonical ensemble (NVE) to preserve the intrinsic atomic dynamics near the friction interface.

To systematically investigate the effects of processing parameters on interfacial atomic diffusion and lattice structural evolution during friction, a series of simulations was conducted with different indentation depths, sliding velocities, and temperatures. The indentation depth was varied at four levels: 0.25, 0.50, 0.75, and 1.00 nm. The sliding velocity was set to 2, 3, 4, 5, and 6 Å/ps, corresponding to 200, 300, 400, 500, and 600 m/s, respectively. It should be noted that these sliding velocities are considerably higher than those typically employed in practical friction-based machining experiments. However, direct quantitative comparison is difficult because of the substantial differences in spatial and temporal scales between MD simulations and experiments. The relatively high velocities in MD simulations are mainly adopted to achieve sufficient atomic-scale deformation and interfacial evolution within the limited simulation time. The simulation temperature was varied from 297 K to 897 K with an interval of 100 K (i.e., 297, 397, 497, 597, 697, 797, 897, and 997 K). The detailed simulation parameters are summarized in Table 1.

## 3. Results and Discussion

### 3.1. Analysis of the Friction Process

The atomic diffusion and lattice evolution behavior at the friction interface during the titanium-induced frictional removal of SiC were analyzed using OVITO (OVITO 3.15.4) [32]. To quantify the number of diffused atoms, the interfacial diffusion layer was first selected based on the spatial distribution and atomic positions at the Ti/SiC interface. The atoms that crossed the original Ti/SiC interface and entered the opposite material region were then identified as diffused atoms, and their numbers were counted separately for Ti, Si, and C atoms. Figure 2a illustrates the selected cross-sectional region used for analyzing the subsurface lattice evolution during friction. The cross-section is perpendicular to the y-axis and located at the center of the SiC substrate along the y-direction, with a thickness of 10 Å. As shown in Figure 2b, significant interfacial atomic diffusion occurs between the titanium slider and SiC substrate. Ti atoms diffuse into the SiC lattice, while Si and C atoms migrate into the Ti lattice. This interdiffusion behavior can be attributed to the high chemical activity of titanium. Under frictional contact conditions, Ti atoms can react with Si and C atoms to form titanium silicide and titanium carbide phases (e.g., TiSi_2_ and TiC) [33], which promote atomic migration and interfacial atomic mixing between Ti and SiC. Furthermore, as shown in Figure 2c,d, pronounced amorphization occurs in both the surface and subsurface regions of SiC after titanium friction. For crystalline materials, friction-induced amorphization represents a severe lattice disordering process and is commonly associated with structural degradation and material removal. Therefore, the MD simulation results demonstrate that titanium friction can effectively induce the removal of SiC through the combined effects of interfacial atomic diffusion and amorphization-assisted structural transformation.

**Figure 3:**
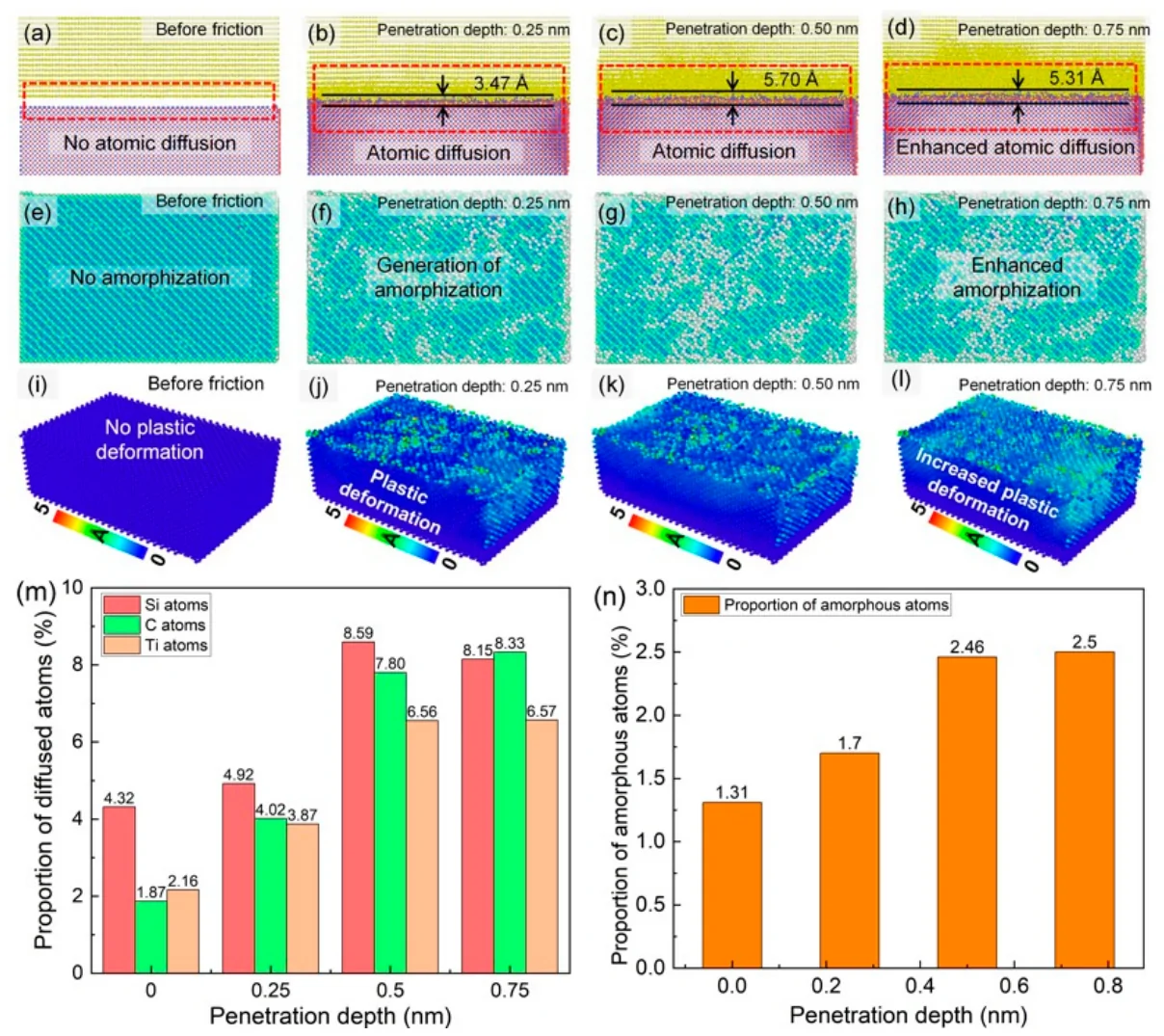
Atomic diffusion at the Ti/SiC friction interface and lattice evolution of the SiC surface during titanium indentation (Si-terminated surface, temperature: 297 K, sliding velocity: 4 Å/ps). (**a**–**d**) Atomic diffusion behavior at the friction interface at different indentation stages, (**e**–**h**) lattice structures of the SiC friction surface at different indentation stages, (**i**–**l**) atomic displacement distributions on the SiC friction surface at different indentation stages, (**m**) variation in the number of diffused atoms across the friction interface with indentation depth, and (**n**) variation in the number of amorphous atoms in the SiC surface region with indentation depth.

As shown in Figure 3a–h, no obvious atomic diffusion occurs at the Ti/SiC interface before indentation, and the SiC lattice maintains a well-ordered crystalline structure without apparent subsurface amorphization or structural damage. With the increasing indentation depth of the titanium slider, Si atoms from the surface region of SiC gradually diffuse into the titanium lattice, while Ti atoms simultaneously migrate into the SiC lattice. The thickness of the interfacial diffusion layer increases from 3.47 Å to 5.31 Å, accompanied by a progressive increase in the amorphization degree of the SiC surface region. As quantitatively illustrated in Figure 3m,n, when the indentation depth increases from 0 to 0.75 nm, the fraction of Ti atoms diffused at the frictional interface increases from 2.16% to 6.57%, the fraction of diffused Si atoms rises from 4.32% to 8.15%, and the fraction of diffused C atoms grows from 1.87% to 8.33%. Meanwhile, the number of amorphous atoms within the SiC lattice increases from 614 to 1174, indicating that deeper indentation intensifies interfacial atomic mixing and lattice disordering. Furthermore, according to Figure 3i,l, pronounced atomic displacement and surface deformation occur on the SiC surface during the progressive indentation of the titanium slider. The atomic displacement distributions obtained using OVITO (OVITO 3.15.4) were used to characterize the spatial extent and degree of deformation of the SiC surface. These MD simulation results demonstrate that increasing the indentation depth enhances the normal load, promotes interfacial atomic diffusion and lattice deformation, and consequently facilitates friction-induced material removal.

**Figure 4:**
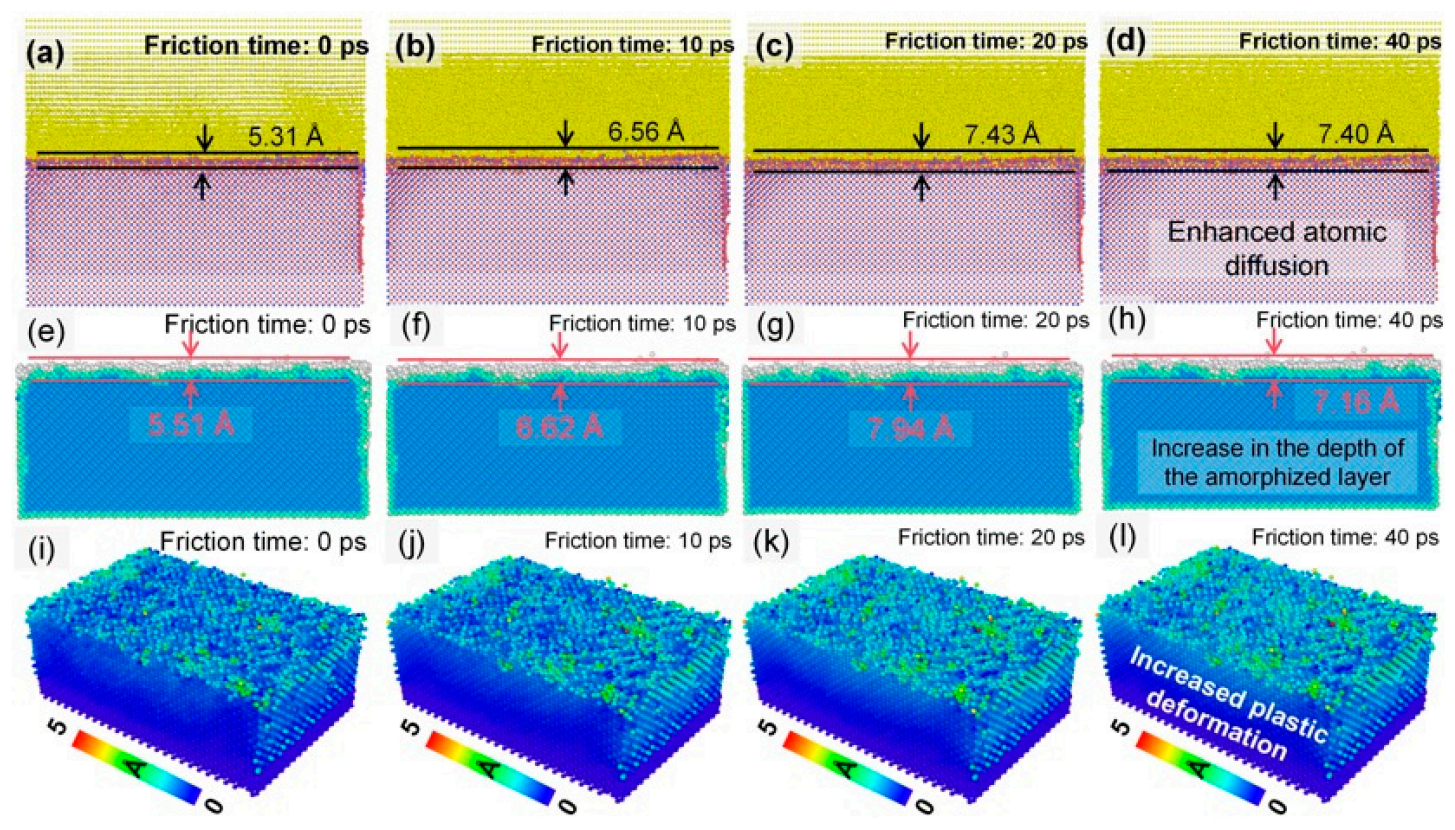
Evolution of lattice structures and atomic displacement on the SiC surface during the friction process (C-terminated surface, temperature: 297 K, sliding velocity: 4 Å/ps, indentation depth: 0.75 nm). (**a**–**d**) Lattice structures of the friction surface at different sliding times, (**e**–**h**) atomic displacement distributions on the SiC surface at different sliding stages, (**i**–**l**) atomic displacement of the friction surface at different friction moments.

As shown in Figure 4a–h, as the friction time increases from 0 ps to 40 ps, atomic diffusion at the SiC frictional interface and the degree of amorphization in the friction subsurface are both significantly enhanced. The thickness of the atomic diffusion layer increases from 5.31 Å to 7.40 Å, and the depth of the subsurface amorphous layer rises from 5.51 Å to 7.16 Å. This evolution indicates that titanium-induced friction promotes structural degradation of the SiC surface layer through interfacial atomic interactions. The underlying removal mechanism is primarily attributed to the combined effects of interfacial atomic diffusion and friction-induced amorphization caused by mechanical shear loading. Furthermore, as shown in Figure 4i–l, the atomic displacement and associated surface deformation of the SiC progressively increase with continued sliding, and residual deformation remains after the friction process. This phenomenon suggests that atomic displacement within the SiC surface layer continuously accumulates during friction, resulting in irreversible lattice distortion and structural modification. The MD simulation results confirm that titanium-induced friction can effectively induce deformation and removal of the SiC surface layer through the synergistic effects of mechanical deformation, atomic migration, and amorphous structure formation.

### 3.2. Effect of Si-Terminated and C-Terminated Surfaces

**Figure 5:**
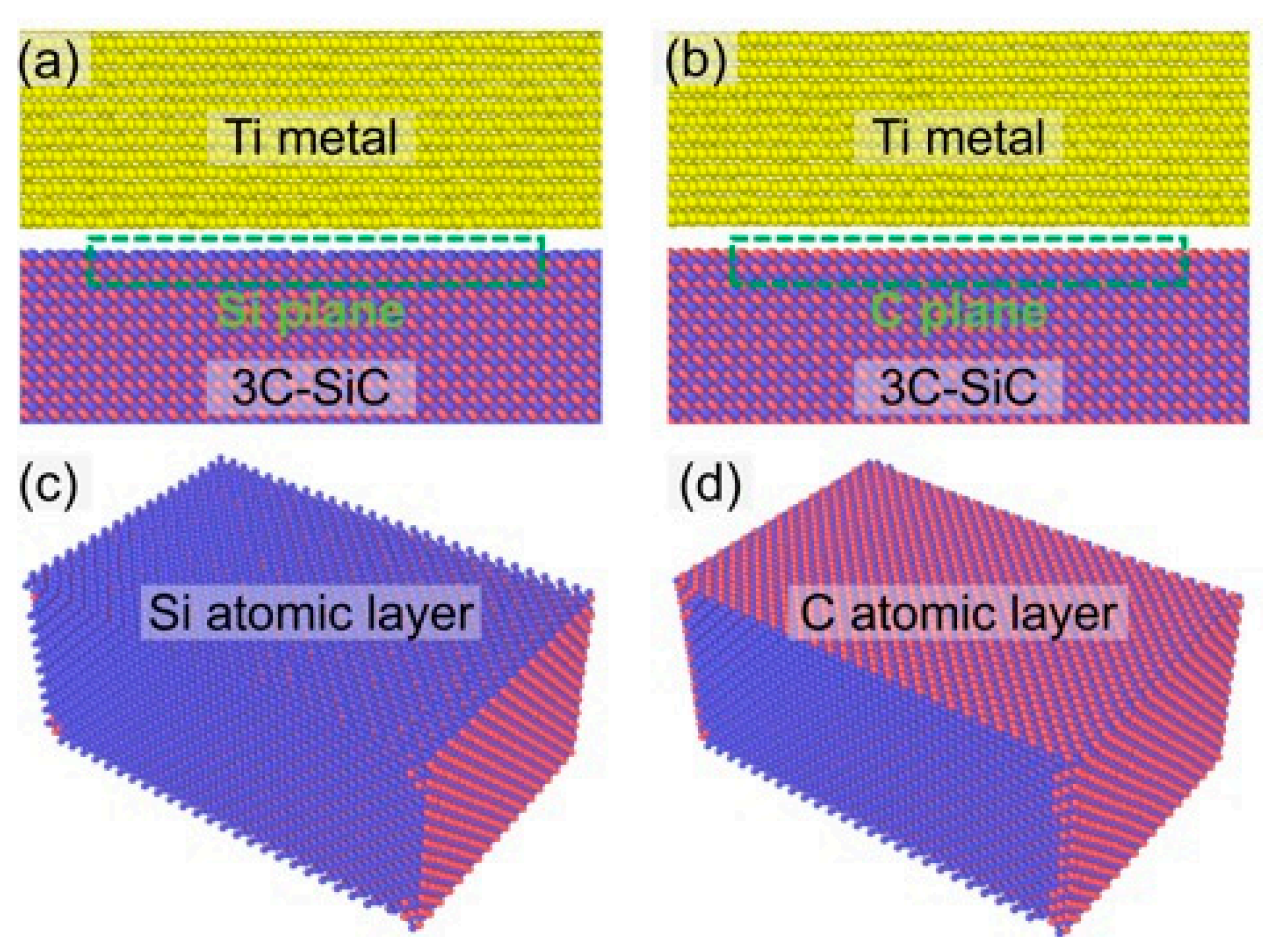
MD simulation models for titanium friction on the Si-terminated and C-terminated SiC surfaces. (**a**) MD model of Ti friction on the Si-terminated surface, (**b**) MD model of Ti friction on the C-terminated surface, (**c**) atomic arrangement of the Si-terminated surface, and (**d**) atomic arrangement of the C-terminated surface.

Due to the unique atomic arrangement of SiC crystals, two distinct surface configurations, namely the Si-terminated surface and the C-terminated surface, exist on the SiC substrate. These two surfaces exhibit significantly different physicochemical properties, which may strongly influence the friction-induced removal behavior. Therefore, MD models with different surface terminations were established, as shown in Figure 5a,b, to systematically investigate the effect of surface orientation on the titanium-induced frictional removal of SiC. As illustrated in Figure 5c, the outermost atomic layer of the Si-terminated surface is composed of Si atoms, whereas the outermost layer of the C-terminated surface consists of C atoms (Figure 5d). Although both surfaces possess the same SiC crystal structure, their surface chemical activity and atomic bonding characteristics are significantly different. In this study, identical titanium sliders and friction conditions were applied to both surfaces to clarify the influence of surface termination on the friction-induced removal mechanism. It should be noted that these idealized surface terminations are not intended to exactly reproduce experimentally prepared SiC surfaces, which usually contain mixed terminations and surface defects. Instead, they were adopted as representative atomic models to isolate the effect of surface chemistry on Ti/SiC interfacial interactions and friction-induced material removal.

**Figure 6:**
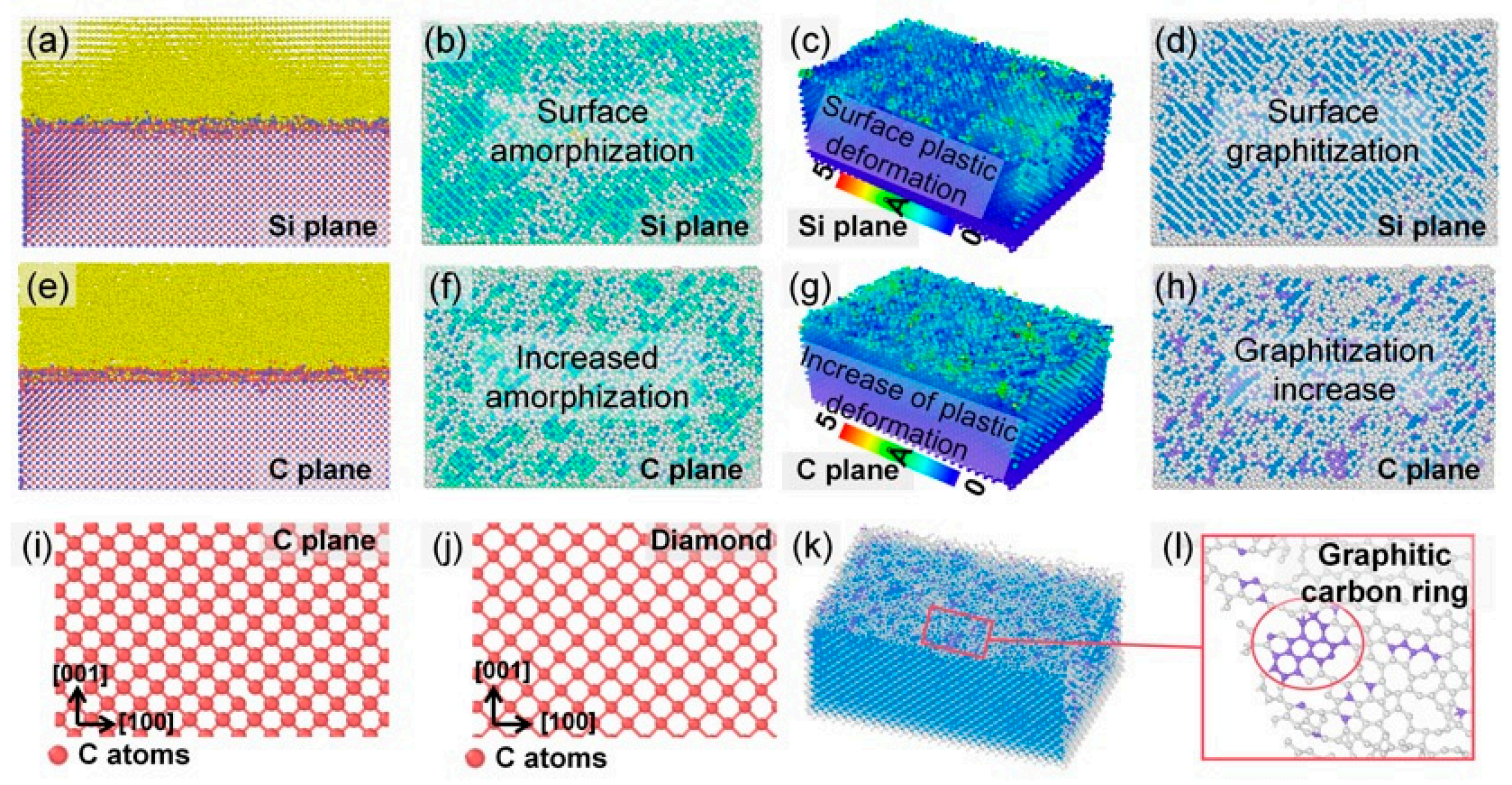
Atomic-scale analysis of lattice evolution during titanium friction on the Si-terminated and C-terminated SiC surfaces (temperature: 297 K, sliding velocity: 4 Å/ps, indentation depth: 0.75 nm, sliding time: 40 ps). Si-terminated surface: (**a**) atomic diffusion at the friction interface, (**b**) amorphous structure of the friction surface, (**c**) atomic displacement distribution, and (**d**) graphite-like structures. C-terminated surface: (**e**) atomic diffusion at the friction interface, (**f**) amorphous structure of the friction surface, (**g**) atomic displacement distribution, and (**h**) graphite-like structures. (**i**) Atomic arrangement of the C-terminated (100) surface of 3C-SiC, (**j**) atomic arrangement of the diamond (100) surface, and (**k**,**l**) local graphitic structures formed on the SiC friction surface.

Figure 6a,e shows that under identical friction conditions and sliding durations, the C-terminated surface exhibits more pronounced atomic diffusion across the Ti/SiC interface compared with the Si-terminated surface. The diffusion layer generated during titanium friction is also significantly deeper on the C-terminated surface. Furthermore, as shown in Figure 6b,f, the number of amorphous atoms on the C-terminated surface after friction reached 2041, which is considerably higher than that on the Si-terminated surface (1429). This indicates that the C-terminated surface undergoes more severe friction-induced amorphization. The atomic displacement distributions shown in Figure 6c,g further demonstrate that the C-terminated surface experiences larger atomic displacements than the Si-terminated surface after titanium friction, indicating more pronounced surface deformation and structural modification. This behavior can be attributed to the stronger chemical affinity between the titanium and carbon atoms. Titanium is a typical carbon-soluble transition metal, and the formation of Ti–C bonds is energetically more favorable than Ti–Si interactions. Consequently, carbon atoms on the C-terminated surface can more readily react with titanium atoms, leading to enhanced atomic migration and interfacial chemical interaction.

The intrinsic mechanical properties of the C-terminated surface do not directly explain this enhanced removal behavior. Although the C-terminated surface contains stronger C–C bonds than the Si–Si bonds associated with the Si-terminated surface, with average bond energies of approximately 347 and 226 kJ mol^−1^, respectively [34], the chemically active titanium interface plays a dominant role in the removal process. The stronger intrinsic C–C bonding contributes to the structural stability of the C-terminated surface; however, the strong chemical affinity between Ti and C promotes interfacial atomic rearrangement, lattice disordering, and amorphization, thereby facilitating the friction-induced removal of the C-terminated surface.

In addition to atomic diffusion and amorphization, titanium-induced graphitization was also observed on both the Si-terminated and C-terminated surfaces after friction, as shown in Figure 6d,h. The C-terminated surface exhibited a much higher degree of graphitization than the Si-terminated surface. Previous studies have demonstrated that titanium can promote the graphitization of diamond under frictional and thermally activated conditions, where Ti–C chemical interactions, mechanical shear, and frictional heating facilitate the transformation of diamond-like carbon structures into graphitic structures [35]. As shown in Figure 6i,j, the surface atomic arrangement of C atoms in the (100) plane of 3C-SiC exhibits structural similarity to the carbon arrangement in diamond, indicating that titanium may also induce the titanium-catalyzed graphitization of carbon structures within SiC. For the Si-terminated surface, titanium atoms must first penetrate the surface Si atomic layer before interacting directly with the underlying carbon atoms, which increases the difficulty of carbon structural transformation. Therefore, under identical friction conditions, the C-terminated surface exhibits more significant graphitization than the Si-terminated surface. To further verify the occurrence of graphitization on the C-terminated surface, the local atomic arrangement and bonding configuration after friction were analyzed using OVITO. The graphite-like carbon structures were identified from the local atomic configurations, particularly the formation of characteristic hexagonal carbon ring structures, and the number of corresponding graphitized C atoms was counted using OVITO. As shown in Figure 6k,l, hexagonal carbon ring structures characteristic of graphitic carbon can be observed in the friction-induced transformed region, confirming the formation of graphite-like structures. Overall, the enhanced atomic diffusion, amorphization, and graphitization induced by titanium friction synergistically promote the removal of SiC. Consequently, the C-terminated surface exhibits a higher friction-induced removal efficiency than the Si-terminated surface.

### 3.3. Analysis of the Tribochemical Reaction Mechanism

**Figure 7:**
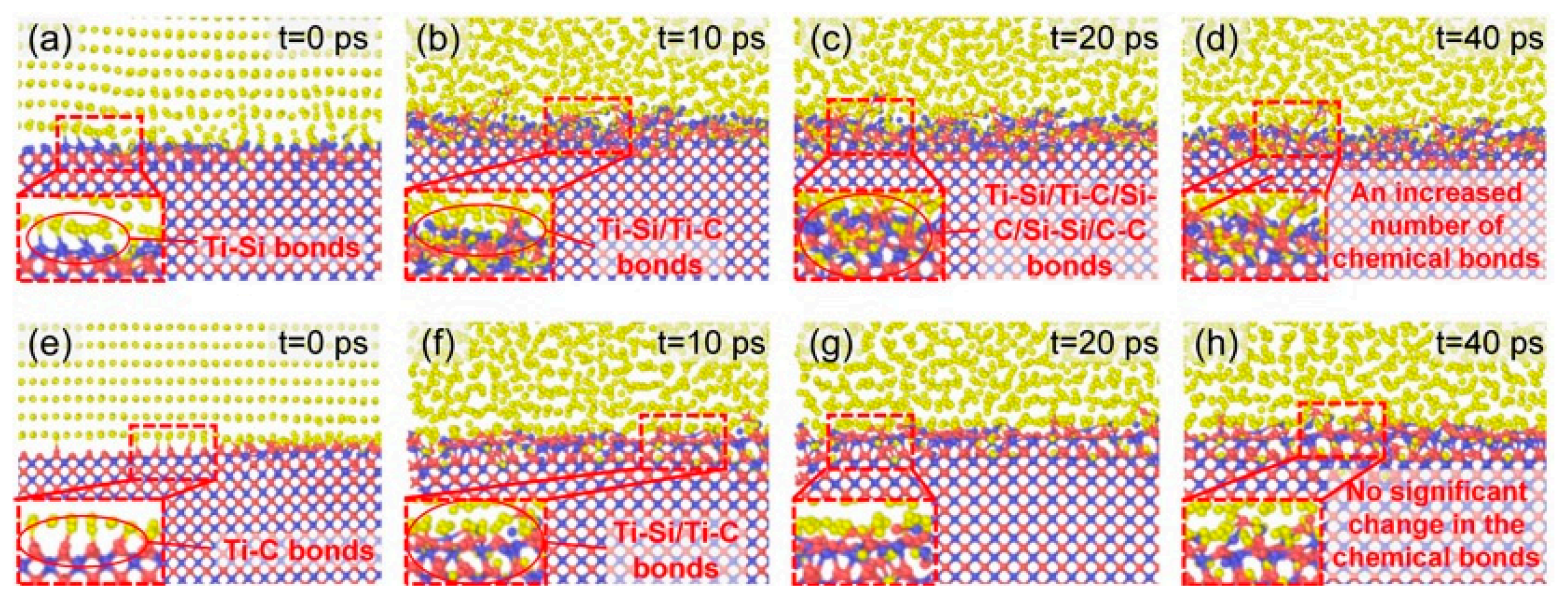
Tribochemical reaction mechanism during titanium friction on the Si-terminated and C-terminated SiC surfaces (temperature: 297 K, sliding velocity: 4 Å/ps). (**a**–**d**) Evolution of interfacial chemical bonds and atomic interactions at different friction stages on the Si-terminated surface, and (**e**–**h**) evolution of interfacial chemical bonds and atomic interactions at different friction stages on the C-terminated surface.

Previous studies have demonstrated that titanium can chemically react with carbon and silicon atoms on the SiC surface under frictional and thermally activated conditions, where mechanical shear and elevated interfacial temperatures promote Ti–C and Ti–Si bond formation and facilitate atomic migration [29,31]. To reveal the atomic-scale tribochemical removal mechanism during the titanium-induced friction of SiC, the evolution of interfacial chemical bonds and atomic interactions on both the Si-terminated and C-terminated surfaces was analyzed using OVITO, and the corresponding results are presented in Figure 7. Ti–C and Ti–Si bonds were identified using the Create bonds modifier based on interatomic distance. The cutoff distances were set to 2.50 Å for Ti–C bonds and 3.00 Å for Ti–Si bonds, respectively. These criteria were consistently applied to characterize the formation and evolution of Ti–C and Ti–Si bonds during friction.

As shown in Figure 7a, during the indentation process, when the Ti atomic layer approaches the Si atomic layer on the Si-terminated SiC surface, chemical bonding occurs between Ti and Si atoms (Equation (4)) [33], leading to the formation of Ti–Si bonds at the interface. With continuous sliding, the number of Ti–Si bonds at the friction interface gradually increases, as shown in Figure 7b. Furthermore, according to Figure 7c,d, a large number of Ti–Si bonds remain at the interface after 30 and 40 ps of friction. Meanwhile, some Ti atoms penetrate into the SiC lattice and interact with C atoms, resulting in the formation of Ti–C bonds (Equation (5)) [33]. The formation of Ti–Si and Ti–C bonds indicates strong interfacial chemical interactions during titanium friction. The newly formed Ti-based bonds exhibit stronger bonding interactions and contribute to the disruption of the stable SiC lattice structure. Under the combined effects of mechanical shear stress and tribochemical reactions, Si atoms on the Si-terminated surface are gradually detached from the SiC lattice, facilitating friction-induced material removal. The chemical bonding evolution during titanium friction on the C-terminated surface is shown in Figure 7e–h. Before sliding, Ti atoms approaching the C-terminated surface also interact with C atoms and form Ti–C bonds at the interface. As the friction process proceeds, the number of Ti–C bonds increases significantly, indicating enhanced interfacial chemical activity. Due to the strong chemical affinity between titanium and carbon, Ti–C bond formation promotes the disruption of C–C and Si–C bonds within the SiC lattice. The formation of these Ti-containing compounds is associated with the strong chemical affinity between the titanium and Si/C atoms. According to the Pauling electronegativity scale, Ti (1.54) has a lower electronegativity than C (2.55) and Si (1.90), resulting in electron transfer from Ti to C/Si and the formation of polar Ti–C and Ti–Si bonds. The stronger electronegativity difference between Ti and C leads to a stronger Ti–C interaction, which explains the enhanced diffusion and structural transformation observed on the C-terminated SiC surface.

(4) Ti+Si→TiSi

(5) Ti+C→TiC

Although the present MD simulations do not directly determine the equilibrium phase composition, the observed Ti–C and Ti–Si bonding configurations indicate strong tribochemical interactions at the Ti/SiC interface. The continuous mechanical activation during friction promotes atomic mixing and increases the probability of Ti reacting with Si and C atoms. Therefore, the formation of Ti–C/Ti–Si bonds should be regarded as an interfacial bonding evolution process that contributes to the disruption of the Si–C network and facilitates material removal.

In addition, although obtaining perfectly C-terminated or Si-terminated SiC surfaces is experimentally difficult, the present comparative analysis provides insight into the fundamental role of surface chemistry during titanium-assisted SiC removal. The enhanced Ti–C interaction revealed on the C-terminated surface suggests that local carbon-rich regions or carbon-dominated surface configurations may significantly influence the tribochemical reactions and removal efficiency in practical SiC machining processes.

## 4. Effect of Processing Parameters

### 4.1. Effect of Indentation Depth

The indentation depth of the metal slider significantly influences the normal load at the friction interface and therefore plays a critical role in controlling the friction-induced removal behavior of SiC. To investigate the influence of indentation depth on the interfacial atomic migration and lattice evolution during the titanium-induced frictional removal of SiC, a series of MD simulations with different indentation depths was performed. Since the Si-terminated surface is the most commonly used crystal orientation in semiconductor and electronic device applications, the Si-terminated SiC surface was selected for investigating the effects of processing parameters.

**Figure 8:**
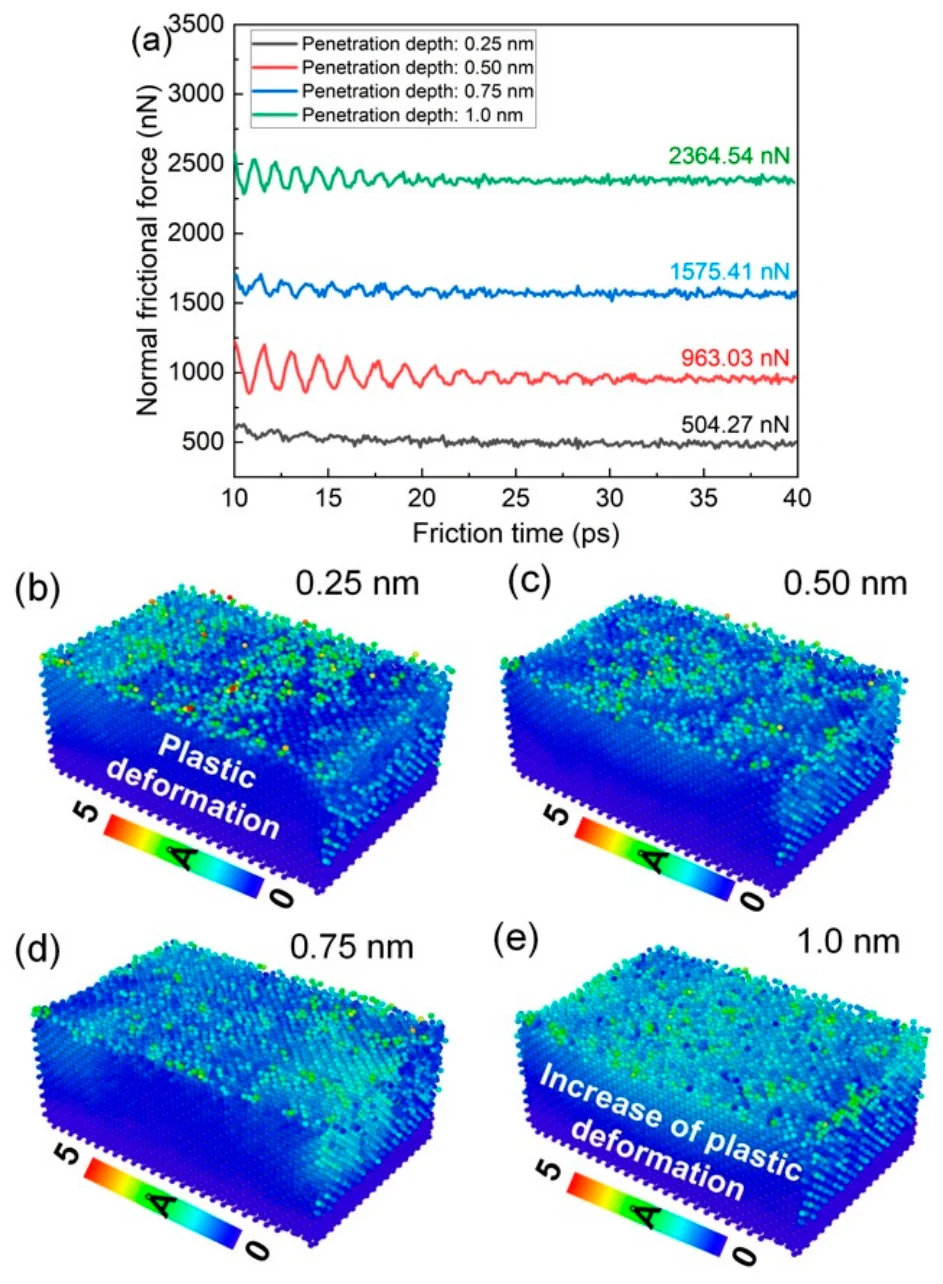
Effect of indentation depth on the normal load and atomic displacement of the SiC friction surface (temperature: 297 K, sliding velocity: 4 Å/ps, sliding time: 40 ps). (**a**) Normal load–time curves under different indentation depths, and (**b**–**e**) atomic displacement distributions on the SiC friction surface under different indentation depths.

The simulations were conducted at 297 K with a sliding velocity of 400 m/s, and the variations in normal load and atomic displacement on the friction surface under different indentation depths are presented in Figure 8. As shown in Figure 8a, when the indentation depth increased from 0.25 nm to 1.0 nm, the normal load at the friction interface increased significantly from 504.27 nN to 2364.54 nN. Meanwhile, as shown in Figure 8b–e, the atomic displacement on the SiC friction surface increased progressively with increasing indentation depth. These results indicate that increasing the indentation depth enhances the mechanical interaction between the Ti slider and SiC substrate by increasing the contact load, thereby promoting plastic deformation of the SiC surface.

**Figure 9:**
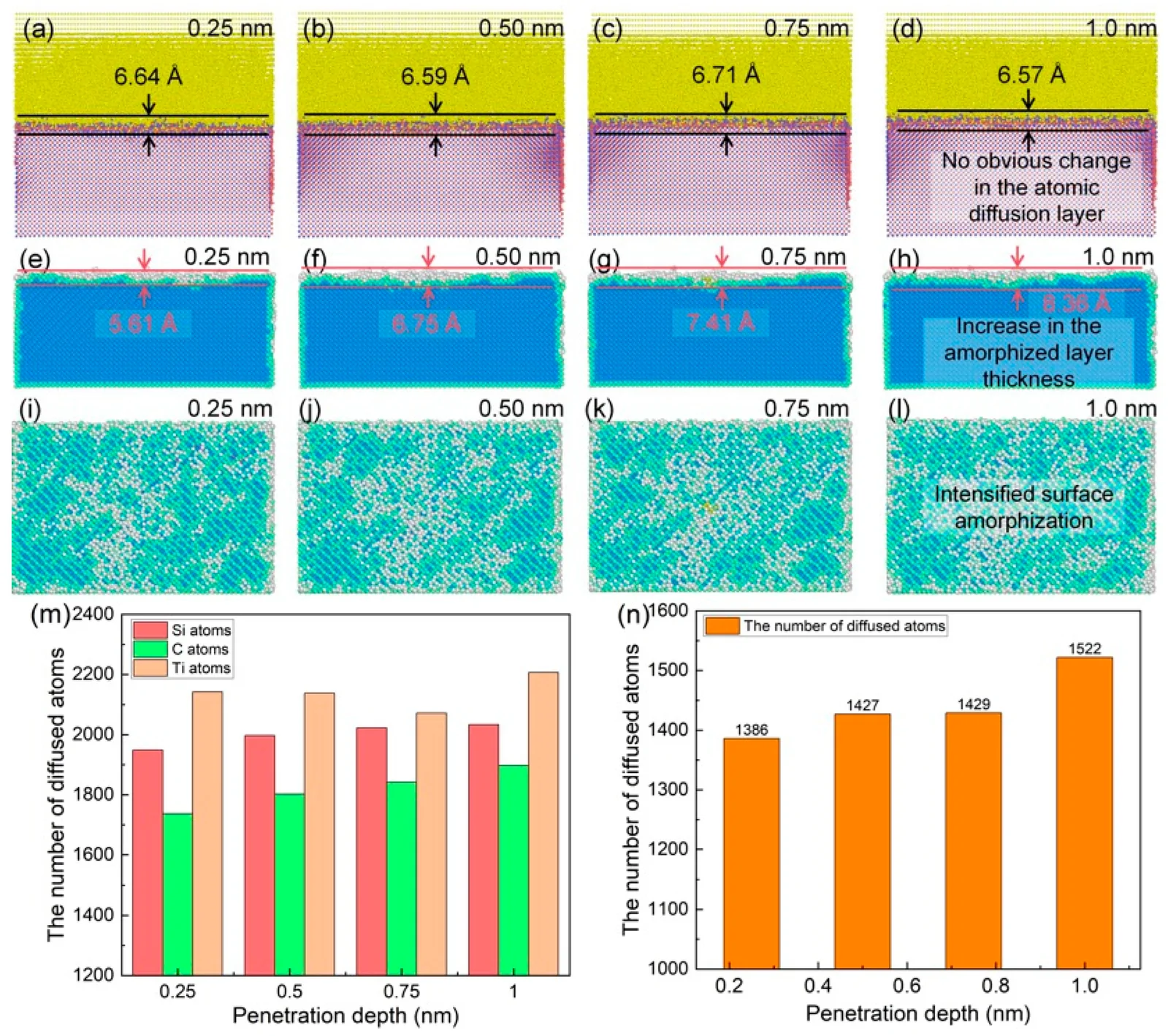
Lattice structures of the SiC friction surface and atomic diffusion behavior at the Ti/SiC interface under different indentation depths (temperature: 297 K, sliding velocity: 4 Å/ps, sliding time: 40 ps). (**a**–**d**) Atomic diffusion behavior at the friction interface, (**e**–**h**) amorphization phenomenon in the friction subsurface. (**i**–**l**) Lattice structures of the friction surface, (**m**) variation in the number of diffused atoms at the friction interface with indentation depth, and (**n**) variation in the number of amorphous atoms with indentation depth.

The lattice evolution and interfacial atomic diffusion behavior under different indentation depths are further analyzed in Figure 9. As shown in Figure 9a–d, increasing the indentation depth slightly enlarged the atomic diffusion region at the Ti/SiC interface. The thickness of the diffusion layer increased from 7.4 Å to 8.7 Å, and the numbers of diffused Si, C, and Ti atoms also showed a slight increase with increasing indentation depth, as presented in Figure 9m. This indicates that indentation depth has a limited influence on interfacial atomic diffusion, although deeper indentation slightly enhances atomic mixing due to the increased contact pressure. In contrast, the influence of indentation depth on amorphization was much more significant. As shown in Figure 9e–h, the amorphous region within both the surface and subsurface layers of SiC gradually expanded with increasing indentation depth. The thickness of the subsurface amorphous layer increased from 5.61 Å to 8.36 Å as the indentation depth increased from 0.25 nm to 1.0 nm. Furthermore, according to Figure 9n, the number of amorphous atoms increased from 1386 to 1522, demonstrating that deeper indentation promotes friction-induced lattice disordering and structural transformation.

The MD simulation results reveal that increasing the indentation depth mainly enhances SiC removal by increasing the mechanical load and inducing stronger plastic deformation rather than significantly accelerating interfacial atomic diffusion. Although the increase in indentation depth only slightly affects the degree of atomic interdiffusion, the resulting increase in normal load substantially promotes lattice distortion, amorphization, and surface structural degradation, thereby improving the friction-induced removal efficiency of SiC.

### 4.2. Effect of Sliding Velocity

The sliding velocity is an important parameter governing the friction coefficient, interfacial temperature, and atomic interaction behavior during tribological processes. To investigate the influence of sliding velocity on the lattice evolution and atomic migration behavior during the titanium-induced frictional removal of SiC, a series of MD simulations with different sliding velocities were performed on the Si-terminated SiC surface.

**Figure 10:**
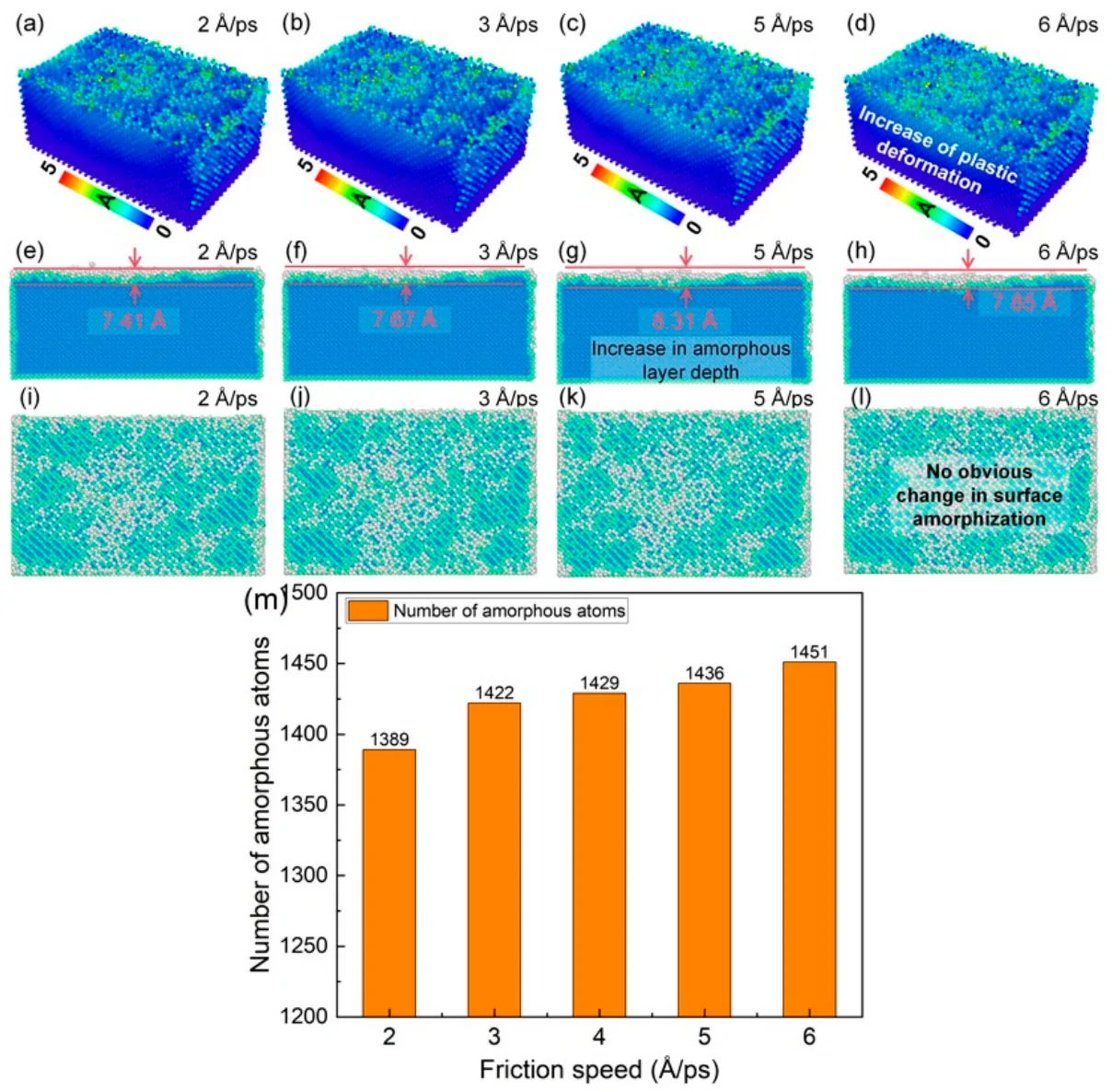
Atomic displacement and lattice evolution of the SiC friction surface under different sliding velocities (temperature: 297 K, indentation depth: 0.75 nm, sliding time: 40 ps). (**a**–**d**) Atomic displacement distributions on the SiC friction surface under different sliding velocities, (**e**–**l**) lattice structures of the SiC surface and subsurface regions under different sliding velocities, and (**m**) variation in the number of amorphous atoms on the SiC friction surface with sliding velocity.

The simulations were conducted at room temperature (297 K) with a constant indentation depth of 0.75 nm. The atomic displacement and lattice evolution of the SiC friction surface under different sliding velocities were analyzed, as shown in Figure 10. As presented in Figure 10a–d, the atomic displacement of the SiC friction surface gradually increased as the sliding velocity increased from 2 Å/ps to 6 Å/ps, indicating that higher sliding velocity enhances the plastic deformation of the SiC surface during titanium-induced friction. The lattice evolution of the surface and subsurface regions is shown in Figure 10e–l. With increasing sliding velocity, the amorphization degree of the SiC surface exhibited only a slight variation, whereas the thickness of the subsurface amorphous layer increased from 7.41 Å to 7.85 Å. As shown in Figure 10m, the number of amorphous atoms increased slightly from 1389 to 1451 when the sliding velocity increased from 200 m/s to 600 m/s. These results indicate that increasing the sliding velocity mainly promotes atomic rearrangement and subsurface structural disordering, thereby enhancing the amorphization-assisted removal behavior of SiC.

**Figure 11:**
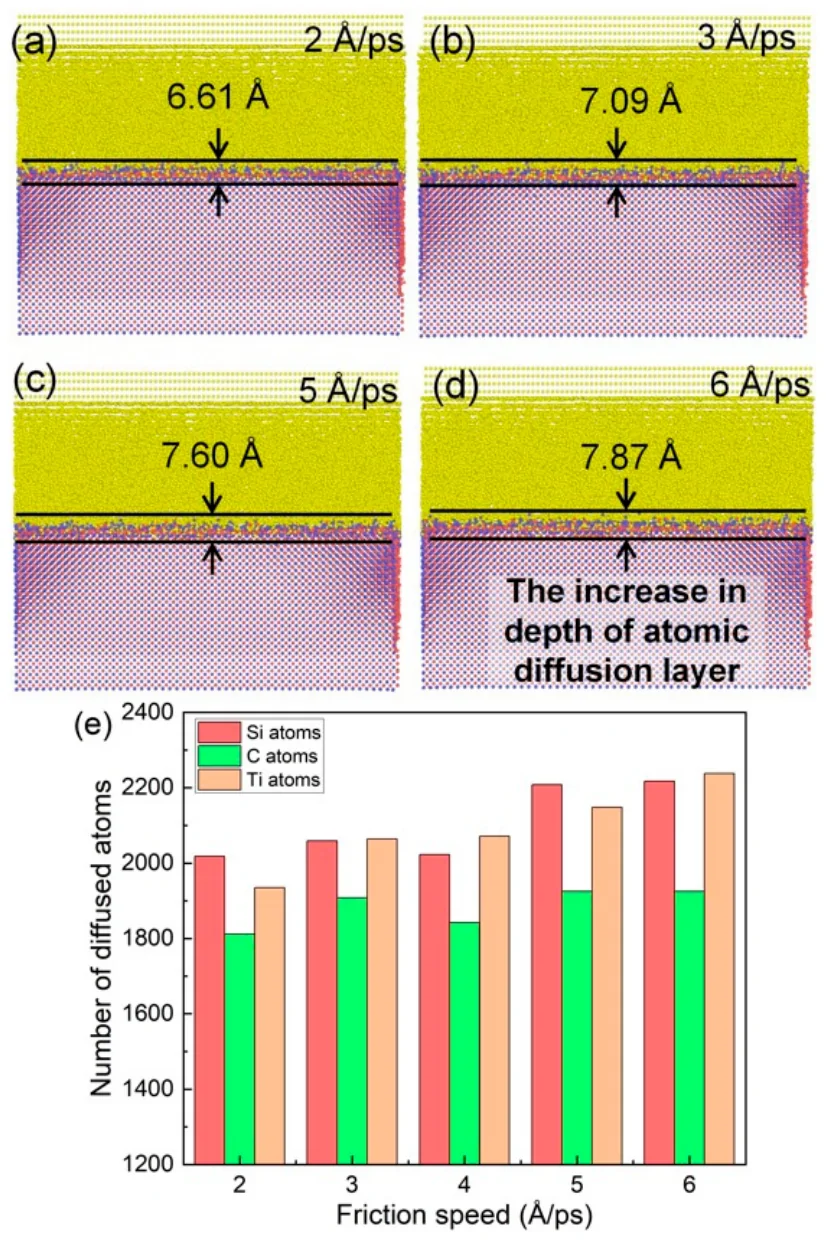
Atomic diffusion behavior at the Ti/SiC friction interface under different sliding velocities. (**a**–**d**) Atomic diffusion configurations at the friction interface under different sliding velocities, and (**e**) variation in the number of diffused atoms at the friction interface with sliding velocity.

The atomic diffusion behavior at the Ti/SiC interface under different sliding velocities is further analyzed in Figure 11. As shown in Figure 11a–d, the thickness of the interfacial diffusion layer remained nearly unchanged with increasing sliding velocity; however, the degree of atomic diffusion became more pronounced. According to Figure 11e, increasing the sliding velocity from 200 m/s to 600 m/s increased the number of diffused Ti, Si, and C atoms from 1935 to 2238, 2018 to 2217, and 1812 to 1925, respectively. These results demonstrate that sliding velocity has a significant influence on the atomic diffusion process at the Ti/SiC interface. A higher sliding velocity enhances atomic thermal activation and interfacial atomic mixing, leading to increased atomic migration, surface plastic deformation, and subsurface amorphization. Therefore, increasing the sliding velocity contributes to improving the friction-induced removal efficiency of SiC by promoting interfacial atomic interaction and structural transformation.

### 4.3. Effect of Temperature

The removal mechanism of the titanium-induced frictional removal of SiC is mainly governed by interfacial atomic diffusion and friction-induced amorphization. Temperature plays a critical role in determining atomic thermal mobility, interfacial chemical activity, and the mechanical response of materials. Therefore, MD simulations were performed to investigate the influence of temperature on atomic diffusion behavior at the Ti/SiC interface and lattice evolution within the SiC subsurface during titanium-induced frictional removal.

#### 4.3.1. Effect of Temperature on the Si-Terminated Surface

**Figure 12:**
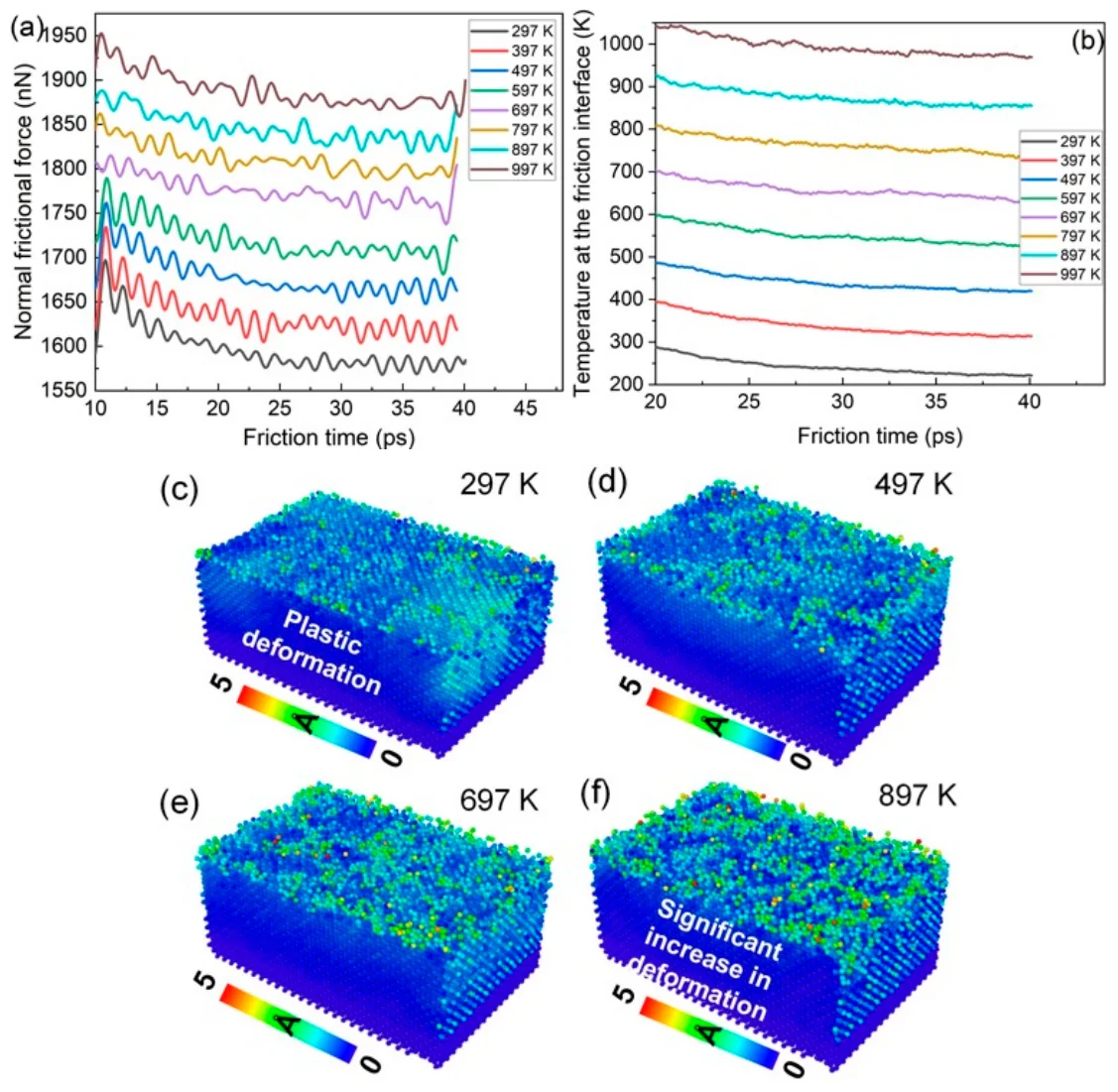
Effect of environmental temperature on the friction behavior of the Si-terminated SiC surface (indentation depth: 0.75 nm, sliding velocity: 4 Å/ps). (**a**) Normal load–time curves under different temperatures, (**b**) interfacial temperature evolution during friction, and (**c**–**f**) atomic displacement distributions on the SiC friction surface under different temperatures.

The variations in friction load and interfacial temperature with sliding time during titanium friction on the Si-terminated SiC surface under different environmental temperatures are presented in Figure 12. The indentation depth and sliding velocity were maintained at 0.75 nm and 400 m/s, respectively. As shown in Figure 12a, when the environmental temperature increased from 297 K to 997 K, the normal friction load between the Ti slider and SiC substrate increased gradually from 1580 nN to 1876 nN. During the simulation, the interfacial temperature became relatively stable after a sliding time of approximately 20 ps. Therefore, the temperature evolution curves within this stable stage were selected to evaluate the effect of environmental temperature on friction-induced thermal behavior. As shown in Figure 12b, increasing the environmental temperature from 297 K to 997 K resulted in a significant increase in the local temperature at the Ti/SiC friction interface, from 236 K to 991 K. This indicates that environmental temperature strongly influences the thermal activation process during the titanium-induced friction removal of SiC. A higher temperature enhances atomic thermal motion and interfacial atomic activity, thereby facilitating atomic migration and structural transformation at the friction interface. Furthermore, as shown in Figure 12c–f, the atomic displacement of the SiC friction surface increased significantly with increasing environmental temperature. The elevated interfacial temperature enhances atomic vibration and mobility, promotes local atomic rearrangement, and intensifies the mechanical deformation of the SiC surface. Consequently, higher temperatures facilitate plastic deformation and structural modification of the SiC friction layer during titanium-induced friction.

**Figure 13:**
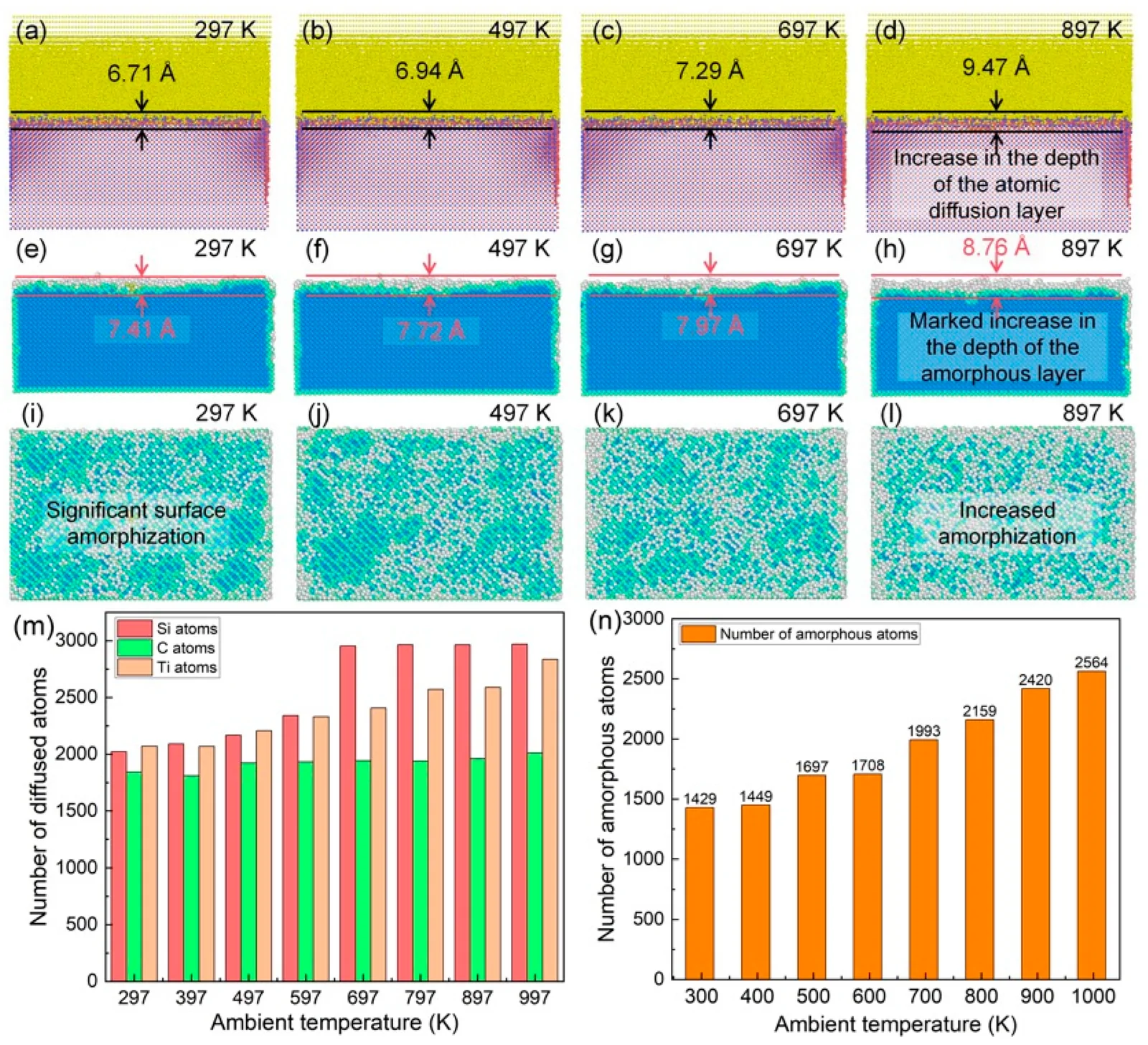
Effect of environmental temperature on atomic diffusion and lattice evolution during titanium friction on the Si-terminated SiC surface (sliding velocity: 4 Å/ps, indentation depth: 0.75 nm, sliding time: 40 ps). (**a**–**d**) Atomic diffusion configurations at the Ti/SiC friction interface under different environmental temperatures, (**e**–**h**) lattice structures of the SiC subsurface region under different environmental temperatures, (**i**–**l**) lattice structures of the SiC friction surface under different environmental temperatures, (**m**) variation in the number of diffused atoms at the friction interface with environmental temperature, and (**n**) variation in the number of friction-induced amorphous atoms with environmental temperature.

The effect of temperature on the titanium-induced frictional removal of SiC is mainly associated with the variation of atomic thermal motion, which regulates atomic diffusion behavior at the Ti/SiC friction interface. The evolution of atomic diffusion at the Ti/SiC interface on the Si-terminated surface under different environmental temperatures is presented in Figure 13.

As shown in Figure 13a–d, the degree of atomic diffusion at the friction interface gradually increased with increasing environmental temperature. The thickness of the interfacial diffusion layer increased from 6.71 Å at room temperature to 9.47 Å at 897 K. Furthermore, as quantitatively shown in Figure 13m, increasing the environmental temperature from 297 K to 997 K resulted in a continuous increase in the number of diffused Ti, Si, and C atoms. The number of diffused Ti atoms increased from 2072 to 2836, while the number of diffused Si and C atoms increased from 2023 to 2970 and 1842 to 2011, respectively. These results demonstrate that elevated temperature enhances atomic thermal activation at the Ti/SiC interface, thereby promoting mutual atomic diffusion between the titanium and SiC. The enhanced diffusion facilitates the migration of Si and C atoms away from the SiC lattice, indicating that higher temperatures contribute to improving the titanium-induced frictional removal of SiC. In addition to atomic diffusion, the amorphization behavior of the SiC surface and subsurface regions is another important indicator for evaluating the friction-induced removal process. The lattice structures of SiC after titanium friction under different environmental temperatures are shown in Figure 13e–l. It can be observed that the subsurface amorphous layer gradually thickened with increasing temperature. Specifically, the thickness of the amorphous layer increased from 7.41 Å at 297 K to 8.76 Å at 997 K, accompanied by a continuous increase in surface amorphization. As presented in Figure 13n, the number of amorphous atoms induced by titanium friction increased significantly from 1429 to 2564 as the environmental temperature increased. The enhanced amorphization indicates more severe lattice disordering and structural transformation within the SiC surface layer, which is closely associated with improved friction-induced material removal efficiency.

Furthermore, Figure 13n reveals a distinct temperature-dependent transition behavior. When the environmental temperature increased from 297 K to 597 K, the number of amorphous atoms increased relatively slowly from 1429 to 1708. However, when the temperature exceeded 597 K, the number of amorphous atoms increased rapidly. This indicates that approximately 597 K represents a critical transition temperature for the titanium-induced frictional removal of SiC. Above this temperature, enhanced thermal activation significantly accelerates atomic diffusion, lattice disordering, and amorphization, resulting in a substantial improvement in the friction-induced removal capability of SiC.

#### 4.3.2. Effect of Temperature on the C-Terminated Surface

The tribological behaviors of the Si-terminated and C-terminated SiC surfaces exhibit significant differences due to their distinct surface atomic configurations. To clarify the influence of temperature on the friction-induced removal behavior of the C-terminated surface, MD simulations were performed to investigate the effects of environmental temperature on atomic diffusion, amorphization, and graphitization during the titanium-induced friction removal of C-terminated SiC.

**Figure 14:**
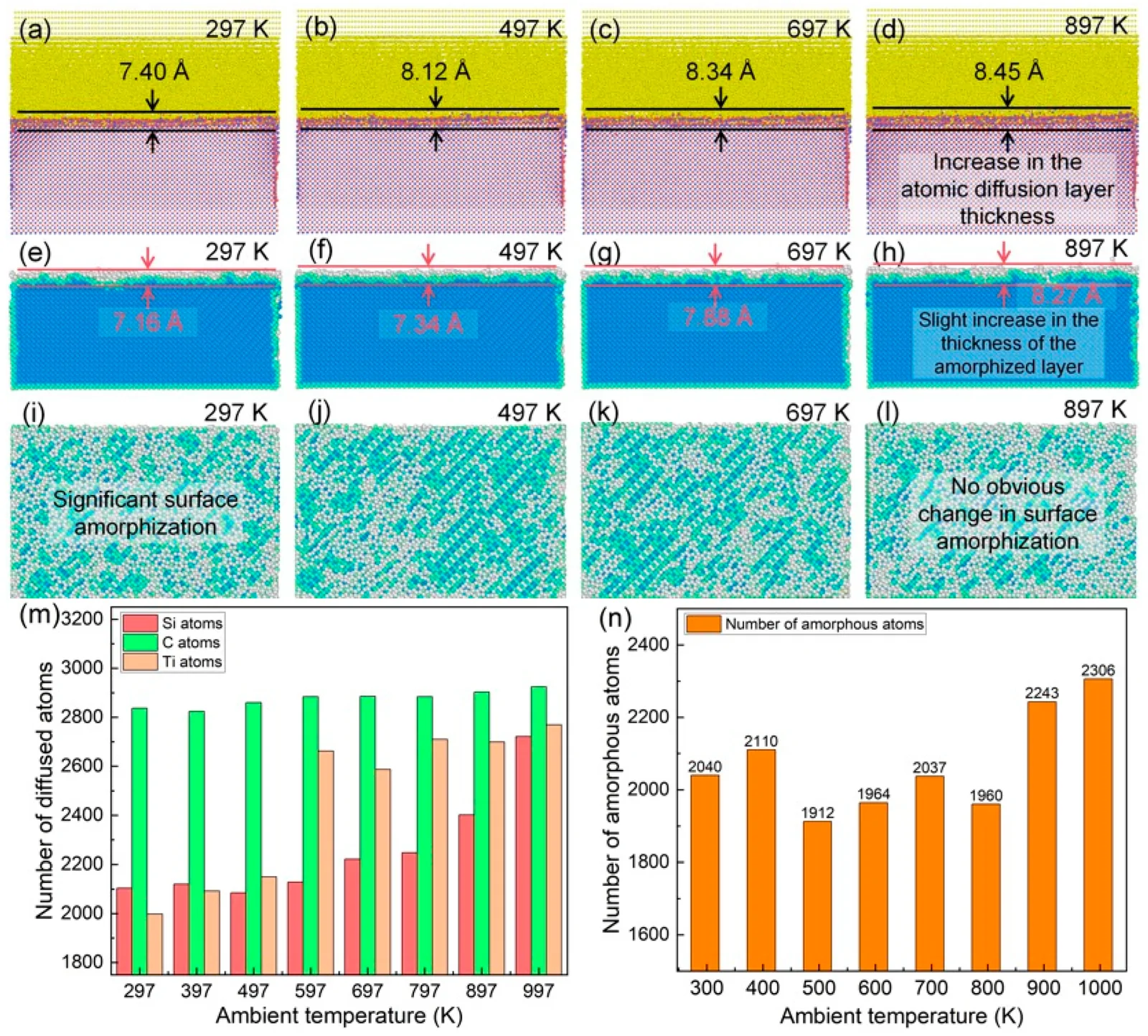
Effect of environmental temperature on atomic diffusion and lattice evolution during titanium friction on the C-terminated SiC surface (sliding velocity: 4 Å/ps, indentation depth: 0.75 nm, sliding time: 40 ps). (**a**–**d**) Atomic diffusion configurations at the Ti/C-SiC friction interface under different environmental temperatures, (**e**–**h**) lattice structures of the SiC subsurface region under different environmental temperatures, (**i**–**l**) lattice structures of the C-terminated friction surface under different environmental temperatures, (**m**) variation in the number of diffused atoms at the Ti/C-SiC interface with environmental temperature, and (**n**) variation in the number of friction-induced amorphous atoms with environmental temperature.

The atomic diffusion behavior at the Ti/C-SiC interface under different environmental temperatures is shown in Figure 14. As observed in Figure 14a–d, the interfacial atomic diffusion became progressively enhanced as the environmental temperature increased from room temperature to 897 K. Under identical friction conditions, the thickness of the diffusion layer increased from 7.40 Å to 8.45 Å. Quantitative analysis in Figure 14m further demonstrates that increasing the temperature from 297 K to 997 K increased the number of diffused Si, C, and Ti atoms from 2104 to 2722, 2837 to 2924, and 1998 to 2769, respectively. These results indicate that environmental temperature plays a significant role in regulating the atomic diffusion behavior during Ti friction on the C-terminated SiC surface. The enhanced thermal activation at elevated temperatures promotes atomic migration and interfacial atomic mixing, thereby accelerating diffusion-assisted material removal. The lattice evolution and amorphization behavior of the C-terminated SiC surface under different temperatures are presented in Figure 14e–l. With increasing environmental temperature, the amorphization degree of both the surface and subsurface regions gradually increases. The thickness of the subsurface amorphous layer increased from 7.16 Å to 8.27 Å as the temperature increased from 297 K to 997 K. According to Figure 14n, the number of friction-induced amorphous atoms increased from 2040 to 2306 with increasing temperature. Furthermore, as shown in Figure 14i–l, higher temperatures resulted in larger atomic displacements within the C-terminated SiC surface, indicating enhanced plastic deformation and improved friction-induced material removal.

Overall, increasing environmental temperature enhances both diffusion-assisted removal and amorphization-assisted removal during titanium friction on the C-terminated SiC surface, thereby improving the removal efficiency of SiC. However, comparison between the Si-terminated and C-terminated surfaces revealed distinct temperature-dependent behaviors. For the Si-terminated surface, increasing temperature resulted in a significant enhancement of surface amorphization. In contrast, the surface amorphization of the C-terminated surface exhibited only a limited increase with temperature. This difference can be attributed to the catalytic graphitization effect of titanium on carbon atoms. During Ti friction on the C-terminated surface, graphitic carbon structures are generated even at relatively low temperatures, resulting in a considerable degree of surface structural disordering before further temperature elevation. Therefore, the amorphization degree of the C-terminated surface remained relatively high across different temperature conditions and showed less pronounced temperature dependence compared with the Si-terminated surface.

**Figure 15:**
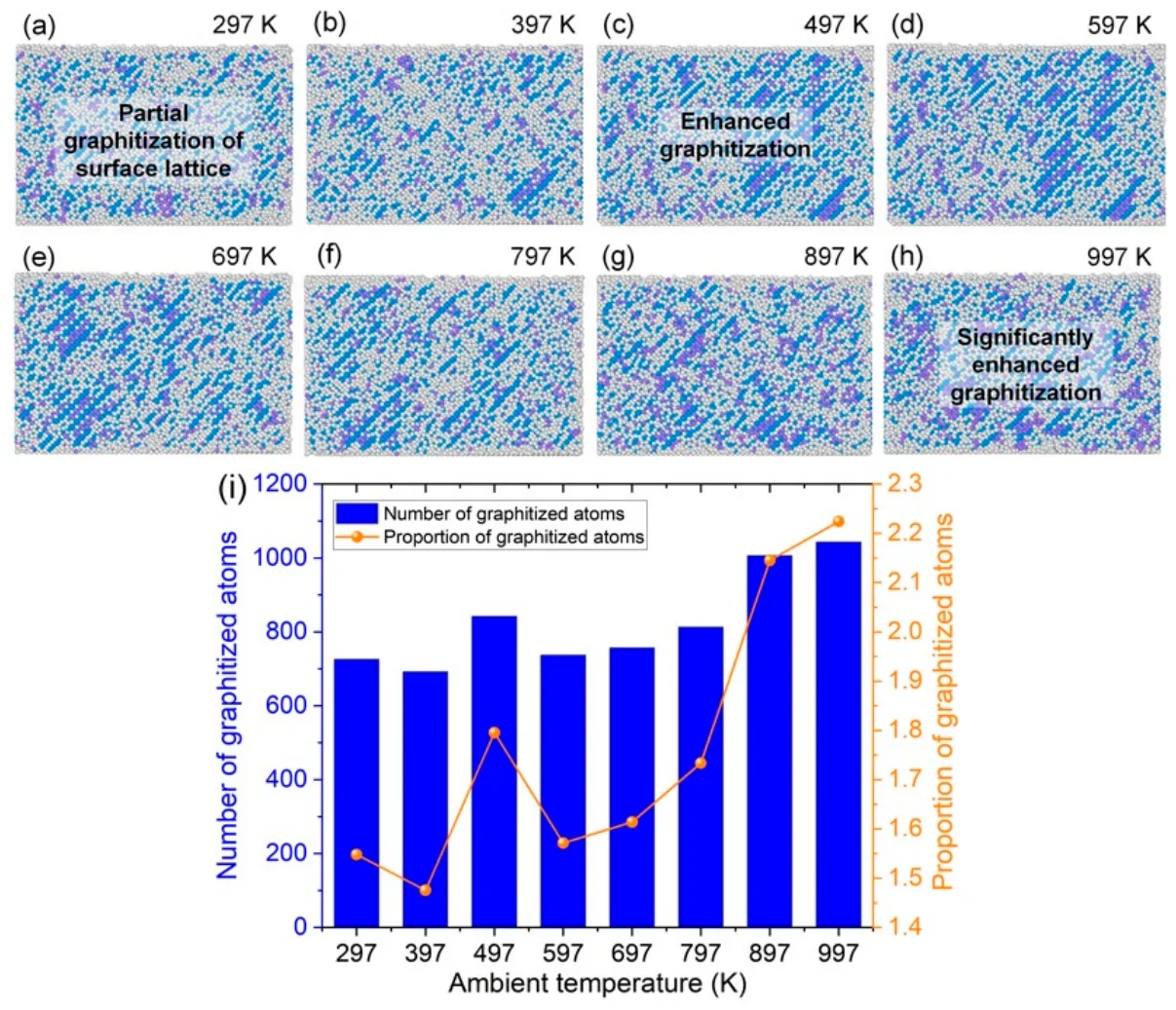
Effect of environmental temperature on graphitic-like lattice structures during friction on the C-terminated SiC surface (sliding velocity: 4 Å/ps, indentation depth: 0.75 nm, sliding time: 40 ps). (**a**–**h**) Graphitic-like lattice structures on the C-terminated SiC friction surface under different environmental temperatures, and (**i**) variation in the fraction of graphitized carbon atoms with environmental temperature.

The effect of environmental temperature on the graphitization behavior of the C-terminated SiC surface during titanium-induced friction was investigated using MD simulations, and the results are presented in Figure 15. It can be observed that graphitization occurred on the C-terminated surface under all investigated temperature conditions. As shown in Figure 15a–h, the number of graphitic carbon structures (highlighted in purple) on the C-terminated surface gradually increased as the environmental temperature increased from 297 K to 997 K, indicating that elevated temperature promotes the graphitization process induced by titanium friction. The quantitative analysis in Figure 15i further revealed that with the continuous increase in ambient temperature, the number of graphitized atoms on the SiC surface rose from 762 to 1043, and the fraction of graphitized atoms increased from 1.55% to 2.22%. These results confirm that increasing environmental temperature enhances the catalytic effect of titanium on carbon graphitization during friction. The elevated temperature provides additional thermal activation energy for carbon atom rearrangement, facilitating the transformation from the original SiC carbon structure into graphitic-like configurations. Therefore, temperature elevation contributes to strengthening the graphitization-assisted removal mechanism of the C-terminated SiC surface.

## 5. Conclusions

In this study, MD simulations were performed to systematically investigate the atomic-scale mechanisms underlying the titanium-induced frictional removal of 3C-SiC and clarify the effects of key processing parameters. The main conclusions are summarized as follows:

(1) During titanium-induced friction on SiC, significant interfacial atomic diffusion occurs, involving the penetration of Ti atoms into the SiC lattice and the migration of Si and C atoms toward the Ti layer. Meanwhile, friction-induced amorphization develops in both the surface and subsurface regions of SiC. The coupled effects of interfacial atomic diffusion, chemical bonding evolution, and amorphization contribute to the degradation and removal of SiC during titanium-assisted friction.

(2) The surface termination of SiC plays a critical role in determining the friction-induced removal behavior. Compared with the Si-terminated surface, the C-terminated surface exhibited stronger chemical interactions with Ti due to the higher affinity between the Ti and C atoms, resulting in enhanced Ti–C bond formation, deeper atomic diffusion, more pronounced amorphization, and more evident carbon structural reconstruction. Consequently, the C-terminated surface demonstrated a higher removal capability during titanium-induced friction.

(3) Increasing the indentation depth significantly increased the normal load at the Ti/SiC interface, thereby enhancing plastic deformation, lattice distortion, and amorphization of the SiC surface. However, the influence of indentation depth on interfacial atomic diffusion is relatively limited, indicating that mechanical loading mainly regulates the structural degradation process rather than the atomic migration behavior.

(4) Increasing the sliding velocity enhances atomic migration and interfacial structural evolution by strengthening the mechanical interaction and thermal activation at the Ti/SiC interface. As a result, higher sliding velocity promotes subsurface amorphization and facilitates friction-induced SiC removal.

(5) Temperature strongly affects the atomic mobility and interfacial reaction activity during titanium-induced friction removal. Elevated temperature promotes interfacial atomic diffusion, lattice disordering, and amorphous layer formation by enhancing atomic thermal activation. For the C-terminated surface, higher temperatures further facilitate the transformation of carbon atoms toward graphitic-like structures, suggesting that thermal activation promotes carbon structural reconstruction during Ti/SiC friction.

Overall, this study reveals the coupled effects of interfacial chemical interactions, atomic diffusion, and structural transformation in the titanium-induced frictional removal of SiC. The findings provide atomic-scale insights into the optimization of titanium-assisted ultra-precision machining strategies for hard and brittle semiconductor materials, particularly SiC, and may also provide useful references for the machining of other hard and brittle semiconductor materials such as GaN, AlN, and diamond.

## Figures and Tables

**Table 1:** Summary of the MD simulation parameters.

Material Properties	Size	Number of Atoms	Lattice Structure
Ti	118 × 80 × 55 Å	2.88 × 10^4^	hcp
3C-SiC	118 × 80 × 50 Å	Si: 2.31 × 10^4^, C: 2.38 × 10^4^	cubic
Potential function	Si–Si, C–C, Si–C: Tersoff/ZBL Ti–Ti, Ti–C, Ti–Si: MEAM		
Time step	1 fs		
Ambient temperature	297–997 K		
Friction speed	Multi-speed, velocity range: 2–6 Å/ps (200–600 m/s)		
Loading direction	Z-axis negative direction		

## Data Availability

The original contributions presented in this study are included in the article. Further inquiries can be directed to the corresponding author.
